# The Week's Work

**Published:** 1911-03-11

**Authors:** 


### The Week's Work.

### THE COST OF INSTALLING AN ALMONER.

Apparently some misapprehension has been caused by a statement in The Hospital of last week that the co-operation between the Leeds Women's Hospital, the General Infirmary, and the Dispensary of the town in the matter of a joint almoner "has been accompanied by a deficit of £980." The exact cost of the almoner's work at the Women's Hospital was £120—the larger figure refers to the increase in the nursing staff and the extension of the work. It is important to make this clear, as the stock excuse made for not reforming the out-patient departments is the alleged cost of paying an almoner, who should devote his or her whole time to the work of investigation of, and report on, all cases seen in the department. This is the more unreasonable because certain institutions—we believe that the Birmingham Hospital and the Radcliffe at Oxford are among them—are employing untrained persons as almoners. Whether this is true of these two hospitals or not, it cannot be emphasised too strongly that the responsible work of a hospital almoner requires careful training, and can successfully be conducted only by an officer of experience and technical knowledge.

### PULMONARY TUBERCULOSIS AT ISOLATION HOSPITALS.

The Macclesfield Health Committee has just sanctioned an interesting scheme for the treatment of cases of pulmonary tuberculosis at the local isolation hospital. It is proposed that a certain number of cases should be received in the now empty smallpox hospital, each case to be kept for a period of about six weeks. During this time the patients will be educated in the principles of hygiene, so far as their special condition is concerned, and will be drilled in preventive methods so that they are likely to become very valuable health missioners in their particular neighbourhoods. The educational and curative advantages of this scheme promise to be very great, and the Medical Officer of Health anticipates that by following such a course for two or more years it will be possible to obtain a material reduction in the mortality rate of phthisis in the Macclesfield district. Under the scheme as approved two nurses and one maid are considered sufficient staff for the small sanatorium which it is intended to start. The total cost of treating fifty patients on this principle is estimated to be just over £366, which would be easily covered by a penny in the pound rate locally. In this schedule of expense the daily cost of feeding the patients is estimated at a shilling per patient, an excess of threepence-halfpenny over the cost per head at the fever hospitals. The excess allowance, in our opinion, is quite justifiable, but on the other hand it seems to us that the provision for structural alterations in the smallpox hospital—estimated at £30—is rather low. It would be unfair to the scheme, since it would to a large extent vitiate the results, to bind the authorities down to that low figure. Such a sanatorium, to be effective, should be very well ventilated, and this would entail considerable modification of the existing window space. We shall watch the results of the scheme with great interest, and congratulate the health authorities of Macclesfield on finding such an excellent use for the tenantless smallpox isolation wards.

### A HOSPITAL CENTENARY.

Last month the hundredth annual general meeting of the Bristol Eye Hospital was held under the chairmanship of Sir Charles D. Cave, Bt., who for more than twenty-five years has held the office of Treasurer to the institution, with the history of which his family has been associated for a hundred years! Such a record of personal service is magnificent; it is a consoling thought in these days that it is not a unique record. Sir Charles is a valuable asset to the Bristol Eye Hospital, and his example has stimulated, and, we feel sure, will continue to stimulate, many to become subscribers to the funds, or at least to take an interest in the work of the century-old institution. It is satisfactory to know that the Eye Hospital is progressing with the times, and that it is free from debt. An increase of £500 per year in the revenue is wanted if this freedom from debt is to be a feature of this year and the succeeding years' reports, for the expenditure is necessarily increasing, since the scope of usefulness of the institution is extending, and since Bristol is not singular in maintaining a steady improvement in methods of treatment at a progressively increasing expense. Such progressive increase in cost of treatment is, taking into consideration the costly accessories which modern ophthalmology demands for its successful practice, inevitable, and friends of the Bristol Eye Hospital will not cavil at it, particularly when they bear in mind that this institution has always been characterised by a rigid economy and by the able way in which its finances have been managed. We congratulate the governors on the centenary meeting, and hope that Bristol will not keep them waiting long before the needed extra £500 per year is secured in the shape of permanent annual subscribers.

### THIS WEEK'S DRUG MARKET.

Business in the drug market has quietened down considerably and few changes in price of any importance have taken place. High quotations are still ruling for cod-liver oil, but the future is uncertain. The values of opium, morphine, and codeine are maintained. Balsam copaiba tends dearer. Cubebs are rather lower. A further advance in the price of Indian hemp is not improbable. Oil of aniseed is dearer, as also is essence of lemon. Ergot of rye continues very dear. Castor oil is slightly cheaper. An advance in the price of cocaine is anticipated. Terebene has been slightly advanced in price and oil of almonds is a little dearer. The demand for menthol is much quieter and prices are rather lower. Oil of male fern is dearer.

